# Peripheral Arterial Disease in Patients Presenting with Acute Coronary Syndrome in Six Middle Eastern Countries

**DOI:** 10.1155/2011/815902

**Published:** 2011-12-18

**Authors:** Hassan A. Al-Thani, Ayman El-Menyar, Mohammad Zubaid, Wafa A. Rashed, Mustafa Ridha, Wael Almahmeed, Kadhim Sulaiman, Ahmed Al-Motarreb, Haitham Amin, Jassim Al Suwaidi

**Affiliations:** ^1^Department of Vascular Surgery, Hamad General Hospital, P.O. Box 3050, Doha, Qatar; ^2^Department of Cardiology and Cardiovascular Surgery, Hamad General Hospital, P.O. Box 3050, Doha, Qatar; ^3^Clinical Medicine, Weill Cornell Medical School, P.O. Box 24144, Doha, Qatar; ^4^Department of Medicine, Faculty of Medicine, Kuwait University, P.O. Box 24923, Kuwait; ^5^Department of Medicine, Mubarak Al-Kabeer Hospital, P.O. Box 43787, Ministry of Health, Kuwait; ^6^Division of Cardiology, Department of Medicine, Adan Hospital, P.O. Box 46969, Ministry of Health, Kuwait; ^7^Sheikh Khalifa Medical City, P.O. Box 51900, Abu Dhabi, UAE; ^8^Royal Hospital, P.O. Box 1331, Muscat, Oman; ^9^Department of Medicine, Faculty of Medicine, Sana'a University, Sana'a, Yemen; ^10^Mohammed Bin Khalifa Cardiac Centre, Manama, Bahrain

## Abstract

To describe prevalence and impact of peripheral arterial disease (PAD) in patients with acute coronary syndrome (ACS), data were collected over 5 months from 6 Middle Eastern countries. Patients were divided into 2 groups (with and without PAD). Out of 6705 consecutive ACS patients, PAD was reported in 177 patients. In comparison to non-PAD, PAD patients were older and more likely to have cardiovascular risk factors. They were more likely to have high Killip class, high GRACE risk score, and non-ST elevation ACS (NSTEACS) at presentation. Thrombolytics, antiplatelet use, and coronary intervention were comparable in both groups. When presented with ST-elevation myocardial infarction (STEMI), patients with PAD had worse outcomes, while in NSTEACS; PAD was associated with higher rate of heart failure in comparison to non-PAD patients. In diabetics, PAD was associated with 2-fold increase in mortality when compared to non-PAD (*P* = 0.028). After adjustment, PAD was associated with high mortality in STEMI (adjusted OR 2.6; 95% CI 1.23–5.65, *P* = 0.01). Prevalence of PAD in ACS in the Gulf region is low. Patients with PAD and ACS constitute a high risk group and require more attention. PAD in patients with STEMI is an independent predictor of in-hospital death.

## 1. Introduction

The prevalence of peripheral arterial disease (PAD) is variable and relatively high in the western world [1–4]. Patients with PAD are at increased risk of coronary, carotid and cerebrovascular atherosclerosis disease, and all-cause mortality [5–8]. This risk is independent of the traditional risk factors such as diabetes mellitus, hypertension, smoking, and obesity [8–10]. PAD is not a static disease and its progression from intermittent claudication to rest pain or gangrene can occur [7–10]. It is possible that the functional impairment in patients with PAD may keep them from ambulating to the point of having angina to the extent that those patients may present with much more advanced coronary atherosclerosis [5]. This risk becomes greater as the severity of PAD increases [7, 8]. Several studies have shown worse prognosis in acute coronary syndrome (ACS) when PAD present in both selected and unselected western population admitted with ACS [1, 3, 5, 11–15]. However, the prevalence and the impact of PAD in patients with acute coronary syndrome in the Middle Eastern countries are limited. The aim of the current study is to study the prevalence of the PAD and to evaluate its impact on the in-hospital mortality and major adverse cardiac events across the ACS population in the Middle Eastern population.

## 2. Methods

For the purpose of the current analysis, data for 6705 consecutive ACS patients was collected from a 6-month prospective, multicenter study of the Gulf Registry of Acute Coronary Events (Gulf RACE) from 6 adjacent Middle Eastern Gulf countries (Bahrain, Kuwait, Qatar, Oman, United Arab Emirates, and Yemen). Patients were recruited from 64 hospitals with the diagnosis of ACS including unstable angina (UA) and non-ST- and ST-elevation myocardial infarction (NSTEMI and STEMI). There were no exclusion criteria and thus all the prospective patients with ACS were actually enrolled. The study received ethical approval from the institutional ethical bodies in all participating countries. Full details of the methods have been published [16, 17]. Data were collected on record forms by the treating physicians. Completed data sheets were sent to the central data processing center, for uniform monitoring and registration. We analyzed patients with peripheral arterial disease (PAD) compared them with those who did not have PAD.

### 2.1. Definitions

Briefly, diagnosis of the different types of ACS and definitions of data variables were based on the American College of Cardiology clinical data standard [18]. For the purpose of this report, ST-segment elevation myocardial infarction and left bundle branch block myocardial infarction were grouped together and called STEMI, whereas merging NSTEMI and unstable angina patients called NSTEACS.

### 2.2. Peripheral Arterial Disease

In addition to well-documented previous history of PAD (i.e., vascular surgery or angioplasty), ankle-brachial index (ABI) of <0.8 in either leg was used as cut point for the presence of PAD. To calculate the ABI ratio, the average systolic blood pressure measurement in the ankle was divided by the average systolic blood pressure measurement in the arm. The mean pressure of the higher arm was used to calculate the ABI separately for each leg.

### 2.3. Statistical Analysis

Patients were divided into 2 groups (with and without PAD). Clinical and biochemical variables, comorbidities and in-hospital medical treatment in ACS patients were analyzed in both groups. Data were presented as proportion or mean ± standard deviation (SD) as appropriate. Differences in categorical variables between respective comparison groups were analyzed using the *χ* ^2^ test. The continuous variables were analyzed using independent-samples *t*-test. The primary end points were analyzed and compared in the different groups using the *χ* ^2^ test. Multivariate logistic regression analysis was carried out after controlling for the relevant variables for the predictors of hospital outcomes. Primary end points included in-hospital reischemia, heart failure (HF), and mortality. The multivariate analysis was adjusted for following potential covariates: age, sex, diabetes mellitus, hypertension, aspirin, heparin, glycoprotein inhibitors, and coronary angiography. Adjusted odds ratios, with accompanying 95% confidence intervals, were reported for the respective categories. Moreover, multivariate logistic regression analysis was carried out after controlling for the relevant variables for the predictors of PAD. All *P* values were two-sided tailed. *P* values of <0.05 were considered significant. All data analyses were carried out using the Statistical Package for Social Sciences version 18 (SPSS Inc. USA). For the purpose of comparing our findings with the western experiences, we selected 4 western originated studies that were conducted on PAD patients presenting with ACS. These studies were SPRINT (Secondary Prevention Study Reinfarction Israeli Nifedipine Trial), GRACE (Global Registry of Acute Coronary Events), PAMISCA (Prevalencia de Afectación de Miembros Inferiores en el paciente con Síndrome Coronario Agudo), and MASCARA (Manejo del Sindrome Coronario Agudo. Registro Actualizado) [1, 11, 14, 15].

## 3. Results

### 3.1. Clinical and Biochemical Profiles

Out of the 6705 patients who were admitted with ACS, PAD was documented in 177 patients (2.6%).  Table 1 shows the baseline characteristics and risk factors of patients with PAD in comparison to non-PAD patients. Patients with PAD were 9 years older (65 ± 11 versus 56 ± 12, *P* < 0.0001), and were more likely to be female (35% versus 24%, *P* < 0.001). PAD patients were also more likely to have diabetes mellitus (69% versus 40%, *P* < 0.001), hypertension (77% versus 50%, *P* < 0.001), dyslipidemia (66% versus 31%, *P* = 0.001), previous history of CAD (79% versus 45%, *P* < 0.001), prior coronary revascularization (35% versus 15%, *P* < 0.001), chronic lung disease (17.5% versus 5%) and renal failure (45% versus 17%, *P* = 0.001). They were less likely to be smokers (32% versus 38%, *P* = 0.001). At presentation with ACS, PAD patients had higher heart rate, Killip class, and GRACE risk score (*P* < 0.001 for all). Mean total cholesterol was lesser in PAD group (4.6 ± 1.5 versus 5.02 ± 2.3, *P* = 0.001) and mean serum triglyceride value was comparable in the 2 groups. NSTEACS was the most frequent diagnosis in PAD patients, whereas STEMI was the predominant diagnosis in non-PAD group.

### 3.2. In-Hospital Treatment Pattern

Table 2 demonstrates the treatment patterns for patients with and without PAD. In regard to on admission therapy, there were no differences between the two groups in the use of oral and intravenous antiplatelet medications, thrombolysis therapy, angiotensin-converting enzyme, or *angiotensinogen*-receptor inhibitors use. Coronary interventions were also comparable in the 2 groups. In PAD group, unfractionated heparin and *β*-blocker were used less frequently (*P* = 0.001), while low molecular weight heparin was more frequently used (*P* = 0.03). At discharge, aspirin and *β*-blocker were less likely used (*P* = 0.001), while clopidogrel and statin were more likely used in PAD group (*P* = 0.03 and 0.01 resp.).

### 3.3. In-Hospital Clinical Outcomes

**Figure 1:**
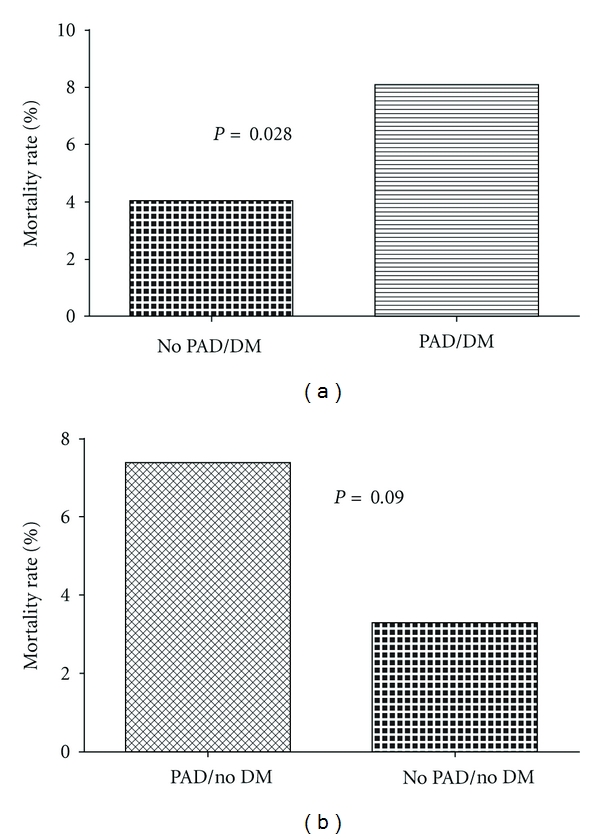
Mortality rate in peripheral arterial disease (PAD) patients presenting with acute coronary syndrome patients and stratified by the diabetic status (DM).

Table 3 shows hospital outcomes in overall, STEMI and NSTEACS patients. In overall ACS and STEMI, all the primary end points were significantly worse in PAD group in addition to the higher percent of bleedings and stroke. In NSTEACS patients, there were no significant differences between the groups except for the high percent of the incidence of heart failure in PAD group (24% versus 16%, *P* = 0.009). Hospital stay was significantly prolonged in PAD group in overall (6.2 versus 5.6 days, *P* = 0.03) and NSTEACS (6.4 versus 5.2 days, *P* = 0.003) patients. In diabetic patients, PAD was associated with 2-fold increase in mortality when compared to non-PAD (8% versus 4%, *P* = 0.028) (Figure 1). There was a significant main effect for PAD (*P* = 0.008) and not for DM (*P* = 0.63) on the mortality; there was no interaction between the two variables (*P* interaction = 0.98).

#### 3.3.1. Multivariate Logistic Regression Analysis (Figure 2)

After adjustment for the important variables (traditional risk factors), the independent predictors for PAD were DM (OR1.9; 95% CI 1.36–2.77), renal failure (OR 2.5; 95% CI 11.79-3.43), smoking (OR 1.8; 95% CI 1.23–2.63), prior CAD (OR 2.3; 95% CI 1.49–3.38), and dyslipidemia (OR 2.3; 95% CI 1.54–3.22). For in-hospital mortality, after adjusting for relevant variables including age, sex, risk factors, treatment, and coronary angiography, PAD was an independent predictor of mortality in STEMI patients (adjusted OR 2.6; 95% CI 1.23–5.65, *P* = 0.01).

## 4. Discussion

The current study demonstrated the prevalence and impact of PAD among patients presenting with ACS who are living in the Middle East. There are several key findings in the present study. Firstly, the prevalence of PAD among ACS patients is low in the gulf region in comparison to the western populations [1, 3, 11–15]. This finding may indicate the underestimation or missed PAD diagnosis in the initial evaluation of ACS patients. Secondly, PAD is a marker of worse baseline cardiovascular risk profile. In overall ACS and STEMI, patients with PAD developed worse in-hospital outcomes in terms of greater rate of death, heart failure, recurrent ischemia, stroke, and major bleeding when compared to their non-PAD counterparts. This was consistent with the previous western studies [1, 11, 14, 15]. Even after adjusting for the potential relevant covariants, PAD was an independent predictor for mortality in STEMI patients in the current study. PAD increased the rate of death almost 3 times in comparison to non-PAD patients. In NSTEACS patients, PAD was associated with significant higher rate of heart failure in comparison to non-PAD. This was consistent with the data from the CRUSADE registry in which PAD was an independent predictor of HF in NSTACS patients [5]. Thirdly, in diabetic patients, PAD increased in-hospital mortality rate twice when compared to their counterpart non-PAD patients. In a recent study, Lafitte et al. [19] reported 3-fold increase in the risk of cardiovascular (CV) events in patients with both PAD and diabetes even after optimization of risk-factor control and medications. This long-term high CV risk was not significant in diabetic patients without PAD.

Previous studies reported that although PAD patients were high risk group, they were less likely to be appropriately treated with evidence-based therapy and this may in part explain the worse outcomes [3, 4, 12, 13]. However, in the current study, there were no significant differences in the management between the 2 groups apart from the fewer use of *β* blockers in PAD group. *β* blockers were not frequently used because of the presence of PAD per se and high percent of chronic lung disease in the PAD group. Fourthly, PAD patients ranked high GRACE risk scoring in patients presenting with ACS; this might be useful simple bedside tool for early risk-stratification of those patients.

Many traditional and emergent CV risk factors are more prevalent in patients with ACS and PAD. Also, certain factors are independent predictors of PAD [10]. However, the associated increased CV risk is independent of those traditional risk factors. The main independent predictors for PAD in the present study included DM, renal failure, prior CAD, and dyslipidemia. The prevalence of PAD in patients with ACS has been reported in several studies in the range of 1% to 39.8% [20–22]. This wide range is influenced by the same factors as the patients without ACS. The prevalence of PAD among patients with CAD varies between studies according to the method of diagnosis [2]. The PAD prevalence ranged between 2.4 in the current study to 39.8 in the PAMISCA study (Table 4). The prevalence in the PAMISCA study was higher than all the other studies and the authors attributed this to the high subclinical PAD patients and the exclusion of patients younger than 40 years of age. In our study, the presence of PAD is probably underestimated in part due to the inclusion criteria (ABI of <0.8), the high prevalence of diabetes mellitus (70%) which can lead to a false elevation of the ankle pressure, and slightly younger age group. The present study reported the highest prevalence of diabetes mellitus when compared to other studies which ranged from 25% to 49.4%. Hypertension and dyslipidemia were higher in patients with PAD when compared with non-PAD patients in all the studies. On the other hand, smoking was lower in patient with PAD compared to non-PAD patients in all the studies with the exception of GRACE study. There were fewer smokers in PAD group in the present study, but interestingly, in multivariate logistic regression analysis, smoking became one of the independent predictors for PAD. Recently, Conen et al. [23] assessed the association of smoking status with PAD in healthy women. The investigators reported that smoking was a potent risk factor for symptomatic PAD and was associated with subclinical inflammation. Also, they observed that smoking cessation substantially reduced risk for PAD, but an increased occurrence of PAD persisted even among former smokers [23].

Data on the prevalence and outcome of Middle Eastern patients with PAD are very limited. Al Zahrani et al. [24] in a cross-sectional hospital-based study (402 patients) in Saudi Arabia reported high prevalence of PAD (ABI <0.9) in elderly high risk patients with either diabetes mellitus (61.4%), chronic renal failure (13.4%), or ischemic heart disease (21.4%) when compared to controls (4.1%). Previously, we conducted a cross-sectional multicenter study in 5 Middle Eastern countries [25]. The study enrolled 1,341 patients who were either (1) with cardiovascular disease (cerebrovascular, ischemic heart disease, and/or peripheral vascular disease) or (2) were at risk of developing cardiovascular disease based on the presence of cardiovascular risk factors. At that study, we reported high prevalence of PAD based on ABI <0.9 (31.5% and 28.2%, resp.). The current study extended these observations and reported for the first time, in our region, the prognostic impact of PAD among ACS patients.

Our data were collected from an observational study which is one of the limitations. However, well-designed observational studies provide valid results and do not systematically overestimate the results compared with the results of randomized controlled trials. Moreover, inconsistent cutoff points for ABI would potentially miss patients with milder PAD leading to potential outcome bias in different studies.

## 5. Conclusion

Prevalence of PAD in ACS in the Gulf region is low. Patients with PAD and ACS are high risk group that require more attention for risk factors and early detection. Certain traditional risk factors are independent predictors for PAD necessitates aggressive preventive measures. PAD in patients with STEMI is an independent predictor for in-hospital death. Detection of PAD in ACS patients might be a useful simple bedside tool for early detection of the risk stratification.

## Figures and Tables

**Figure 2:**
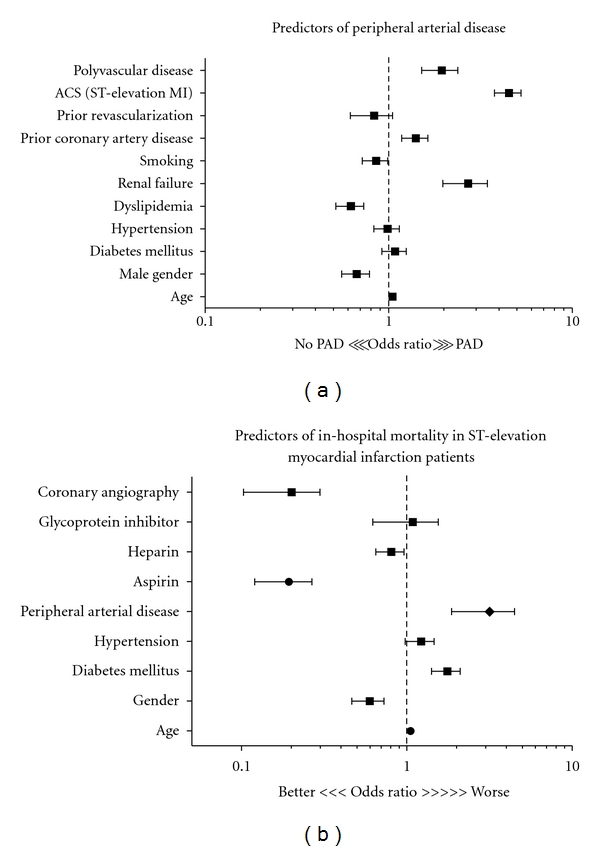
(a) predictors for peripheral arterial disease, (b) clinical predictors for in-hospital mortality in ST-elevation myocardial infarction.

**Table 1:** Clinical and biochemical profiles of patients with acute coronary syndrome.

	Non-PAD (*N* = 6528)	PAD (*N* = 177)	*P* value
Age (mean)	56 ± 12	65 ± 11	0.001
Females (%)	1571 (24)	61 (35)	<.001
Prior coronary artery disease (%)	2929 (45)	140 (79)	<.001
Prior coronary revascularization (%)	987 (15)	62 (35)	<.001
Family history of CAD (%)	873 (13)	29 (16)	0.87
Diabetes mellitus (%)	2622 (40)	123 (70)	<.001
Hypertension	3228 (50)	136 (77)	<.001
Smoking (%)	2491 (38)	56 (32)	<.001
Dyslipidemia (%)	2034 (31)	116 (66)	<.001
Prior aspirin use (%)	2644 (41)	141 (80)	<.001
Renal failure (%)	1091 (17)	80 (45)	<.001
Chronic lung disease (%)	335 (5)	31 (17.5)	<.001
Heart rate (mean, b/min)	86 ± 22	93 ± 27	<.001
SBP (mean, mmgh)	140 ± 30	137 ± 33	0.26
DBP (mean, mmgh)	84 ± 17	78 ± 18	0.001
BMI (mean)	27.6 ± 5	27.4 ± 5	0.77
Ischemic chest pain, *n* (%)	5223 (80)	121 (68)	<.001
Late presentation	801 (31)	13 (28)	0.61
Killip class >I, *n* (%)	1392 (21)	79 (45)	<.001
LV ejection fraction <40% (%)	890 (22)	40 (33)	0.007
NSTEACS	3957 (61)	129 (73)	0.001
STEMI/LBBB	2571(39)	48 (27)	0.001
GRACE risk Scoring			
Low, *n* (%)	2047 (43)	14 (11)	<.001
Medium, *n* (%)	1341 (28)	22 (18)	
High, *n* (%)	1390 (29)	90 (71)	
Biochemical findings			
First blood sugar (mg/dL)	11 ± 10	13 ± 7	<.001
Fasting blood sugar (mg/dL)	8 ± 8	9 ± 4	0.02
Peak troponin (ng/mL)	17 ± 51	7 ±30	0.04
First creatinine (*μ*mol/L)	107 ± 92	163 ±139	<.001
Total cholesterol	5 ± 2	4.6 ± 1.5	<.001
HDL(*μ*mol/L)	1.03 ± 1.2	0.97 ± 0.7	0.22
LDL(*μ*mol/L)	3.3 ± 3	4.6 ± 2	0.001
Fasting triglyceride (*μ*mol/L)	1.9 ± 2	1.8 ± 1.2	0.77
First haemoglobin (mean, gm/L)	14 ± 4	13 ± 6	0.009

PCI: Percutaneous coronary interventions, CAD: coronary artery disease, SBP: systolic blood pressure, DBP: diastolic blood pressure. GRACE: Global registry of acute coronary events.

**Table 2:** Management of patients with acute coronary syndrome.

	No-PAD	PAD	*P* value
On admission medications			
Thrombolysis, *n* (%)	1494 (58)	24 (51)	0.34
Aspirin, *n* (%)	6391(98)	172 (97)	0.49
Clopidogrel, *n* (%)	3499 (54)	106 (60)	0.09
Heparin, *n* (%)	3093 (48)	67 (38)	0.01
LMW Heparin, *n* (%)	3073 (47)	98 (55)	0.03
Gp IIb/IIIa inhibitor, *n* (%)	677 (10)	23 (13)	0.26
*β*-Blocker, *n* (%)	4277 (66)	84 (48)	0.001
ACE/ARB, *n* (%)	4498 (69)	120 (68)	0.74
Coronary angiography (%)	1217 (19)	33 (19)	0.99
PCI, *n* (%)	273 (19.14.2)	7 (19.94)	0.88
Discharge medications			
Aspirin, *n* (%)	6155 (95)	150 (85)	0.001
Clopidogrel, *n* (%)	3204 (49)	101 (57)	0.03
Statin, *n* (%)	5264 (81)	156 (88)	0.01
ACE/ARB, *n* (%)	4950 (76)	132 (75)	0.66
*β*-Blocker, *n* (%)	4939 (76)	107 (61)	0.001
Diuretics, *n* (%)	4561(70)	134 (76)	0.11

**Table 3:** Clinical outcomes in patients with acute coronary syndromes.

	Non-PAD	PAD	*P* value
Overall			
In-hospital death, *n* (%)	233 (4)	14 (8)	0.002
Heart failure, *n* (%)	1044 (16)	55 (31)	0.001
Recurrent ischemia, *n* (%)	580 (10)	24 (14)	0.03
Re-infarction, *n* (%)	151 (2.3)	2 (1.1)	0.29
Major bleeding, *n* (%)	46 (0.7)	6 (3.4)	0.001
Stroke, *n* (%)	45 (0.7)	4 (2.3)	0.01
Hospital stay (mean) (days)	5.6 ± 4.6	6.2 ± 4.2	0.03
STEMI/LBBB			
In-hospital death, *n* (%)	161 (6)	11 (23)	0.001
Heart failure, *n* (%)	430 (17)	24 (50)	0.001
Recurrent ischemia, *n* (%)	233 (9)	9 (19)	.02
Major bleeding, *n* (%)	26 (1)	5 (10)	0.001
Stroke, *n* (%)	29 (1.1)	3 (6.4)	0.001
Hospital stay (mean) (days)	6.14 ± 4.3	5.8 ± 3.7	0.58
NSTEACS			
In-hospital death, *n* (%)	72 (1.8)	3 (2.3)	0.67
Heart failure, *n* (%)	614 (16)	31 (24)	0.009
Recurrent ischemia, *n* (%)	347 (9)	15 (12)	0.27
Major bleeding, *n* (%)	20 (0.5)	1 (0.8)	0.67
Stroke, *n* (%)	16 (0.4)	1 (0.8)	0.52
Hospital stay (mean) (days)	5.17 ± 4.8	6.4 ± 4.4	0.003

**Table 4:** Risk factors and outcomes of peripheral arterial disease in patients presenting with acute coronary syndrome in different studies.

	SPRINT 1994			GRACE 2007			PAMISCA 2008			MASCARA 2009			Gulf RACE 2009		
Patients *n*.	4258			32735			1410			6745			6705		
PAD prevalence	6.3%			7.6%			39.8%			8.8%			2.4%		
	PAD	No PAD	*P* value	PAD	No PAD	*P* value	PAD	No PAD	*P* value	PAD	No PAD	*P* value	PAD	No PAD	*P* value
Age (years)	66 ± 10	62 ± 11	0.01	71	64	0.001	69 ± 11.3	64 ± 11	0.001	70 ± 10	67 ± 10	0.001	65 ± 11	56 ± 12	0.001
Diabetes mellitus	25	20	0.01	38	22	0.001	41.5	30.6	0.001	49.4	28.1	0.001	70	40	0.001
Hypertension	47	39	0.01	72	58	0.001	84.1	76.1	0.001	71.9	58.3	0.001			
Dyslipidemia	—	—	—	58	46	0.001	85.7	83	NS	57	46.4	0.001	66	31	0.001
Smoking	35	36	NS	69	59	0.001	29.9	31.6	NS	21.6	28.3	0.001	32	38	0.001
Hospital outcome															
Death	24	13	0.001	7.2	4.5	0.001	2	0.2	0.001	9.1	4.8	0.001	8	4	0.002
CHF	23	19	NS	—	—	—	15.9	8.4	0.001	—	—	—	31	16	0.001
Re-ischemia/infarction	—	—	—	7.7	8.3	NS	13.7	7.8	0.001	—	—	—	14	10	0.03

SPRINT: Secondary Prevention Study Reinfarction Israeli Nifedipine Trial, GRACE: Global Registry of Acute Coronary Events.

PAMISCA: Prevalencia de Afectación de Miembros Inferiores en el paciente con Síndrome Coronario Agudo, MASCARA: Manejo del Sindrome Coronario Agudo. Registro Actualizado, Gulf RACE: Gulf Register of Acute Coronary Events, CHF: congestive heart failure. [1, 11, 14, 15].
